# Carotenoid β-Ring Hydroxylase and Ketolase from Marine Bacteria—Promiscuous Enzymes for Synthesizing Functional Xanthophylls

**DOI:** 10.3390/md9050757

**Published:** 2011-05-06

**Authors:** Norihiko Misawa

**Affiliations:** Research Institute for Bioresources and Biotechnology, Ishikawa Prefectural University, Suematsu, Nonoichi-machi, Ishikawa 921-8836, Japan; E-Mail: n-misawa@ishikawa-pu.ac.jp; Tel.: +81-76-227-7525; Fax: +81-76-227-7557

**Keywords:** *Paracoccus*, *Brevundimonas*, marine bacteria, ketocarotenoid, functional xanthophyll

## Abstract

Marine bacteria belonging to genera *Paracoccus* and *Brevundimonas* of the α*-Proteobacteria* class can produce C_40_-type dicyclic carotenoids containing two β-end groups (β rings) that are modified with keto and hydroxyl groups. These bacteria produce astaxanthin, adonixanthin, and their derivatives, which are ketolated by carotenoid β-ring 4(4′)-ketolase (4(4′)-oxygenase; CrtW) and hydroxylated by carotenoid β-ring 3(3′)-hydroxylase (CrtZ). In addition, the genus *Brevundimonas* possesses a gene for carotenoid β-ring 2(2′)-hydroxylase (CrtG). This review focuses on these carotenoid β-ring-modifying enzymes that are promiscuous for carotenoid substrates, and pathway engineering for the production of xanthophylls (oxygen-containing carotenoids) in *Escherichia coli*, using these enzyme genes. Such pathway engineering researches are performed towards efficient production not only of commercially important xanthophylls such as astaxanthin, but also of xanthophylls minor in nature (e.g., β-ring(s)-2(2′)-hydroxylated carotenoids).

## 1. Introduction

**Figure 1.**
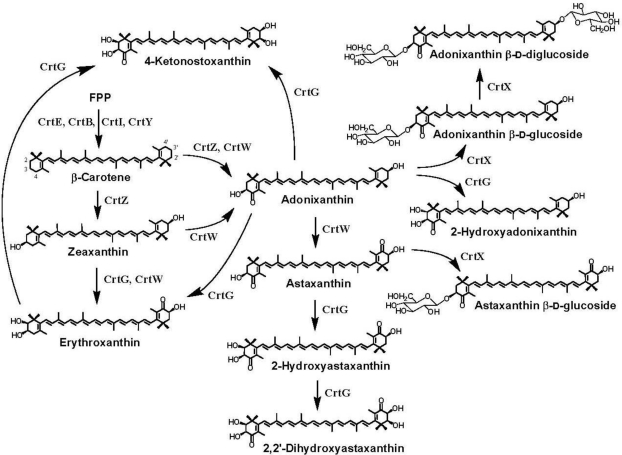
Chemical structures of ketocarotenoids produced in marine bacteria, *Paracoccus* sp. and *Brevundimonas* sp., and feasible functions of the carotenoid biosynthesis enzymes. These bacteria synthesize dicyclic carotenoids. *Paracoccus* sp. and *Brevundimonas* sp. are demonstrated to possess the unique genes *crtX* and *crtG*, respectively, in addition to the common genes, *crtE*, *crtB*, *crtI*, *crtY*, *crtZ*, and *crtW* [10,11].

Many bacteria that have been isolated from marine environments can synthesize a variety of carotenoid pigments [1]. For example, acyclic C_30_-type carotenoic acids were identified in some marine bacteria such as *Planococcus maritimus* [2] and *Rubritalea squalenifaciens* [3]. *Algoriphagus* sp. KK10202C of the *Flexibacteraceae* family, which was isolated from a marine sponge, was found to produce flexixanthin ((3*S*)-3,1′-dihydroxy-3′,4′-didehydro-1′2′-dihydro-β,ψ-caroten-4-one) and deoxyflexixanthin (1′-hydroxy-3′,4′-didehydro-1′2′-dihydro-β,ψ-caroten-4-one) [4], which are C_40_-type monocyclic carotenoids containing one β-end group (β ring) (called monocyclic carotenoids in this review). Other marine bacteria including strain P99-3, which belong to the *Flavobacteriaceae* family, were shown to produce monocyclic carotenoids, myxol ((3*R*,2′*S*)-3′,4′-didehydro-1′,2′-dihydro-β,ψ-carotene-3,1′,2′-triol) and saproxanthin ((3*R*)-3′,4′-didehydro-1′,2′-dihydro-β,ψ-carotene-3,1′-diol), and zeaxanthin ((3*R*,3′*R*)-β,β-carotene-3,3′-diol) [5,6], which are a C_40_-type dicyclic carotenoid containing two β-end groups (called dicyclic carotenids in this review). Marine bacteria belonging to genus *Paracoccus*, *Brevundimonas* or *Erythrobacter* in the α*-Proteobacteria* class have been revealed to synthesize dicyclic carotenoids that are ketolated at the 4(4′)-position(s) (called ketocarotenoids), e.g., astaxanthin ((3*S*,3′*S*)-3,3′-dihydroxy-β,β-carotene-4,4′-dione) and adonixanthin ((3*S*,3′*R*)-3,3′-dyhydroxy-β,β-caroten-4-one) (Figure 1) [7–9].

Among ketocarotenoids, astaxanthin and canthaxanthin (β,β-carotene-4,4′-dione) (specifically the former), are commercially important pigments as nutraceuticals and cosmetics that have anti-oxidation and anti-aging effects as well as colorants in aquaculture, while other ketocarotenoids are likely to have industrial potentials [12–16]. This review focuses on carotenoid β-ring 4(4′)-ketolase (4-oxygenase), carotenoid β-ring 3(3′)-hydroxylase, and carotenoid β-ring 2(2′)-hydroxylase, derived from the marine bacteria that belong to α*-Proteobacteria*, and pathway engineering for the production of functional xanthophylls via the incorporation of these β-ring-modifying enzyme genes.

## 2. Bacterial Strains Producing Ketocarotenoids

**Figure 2.**
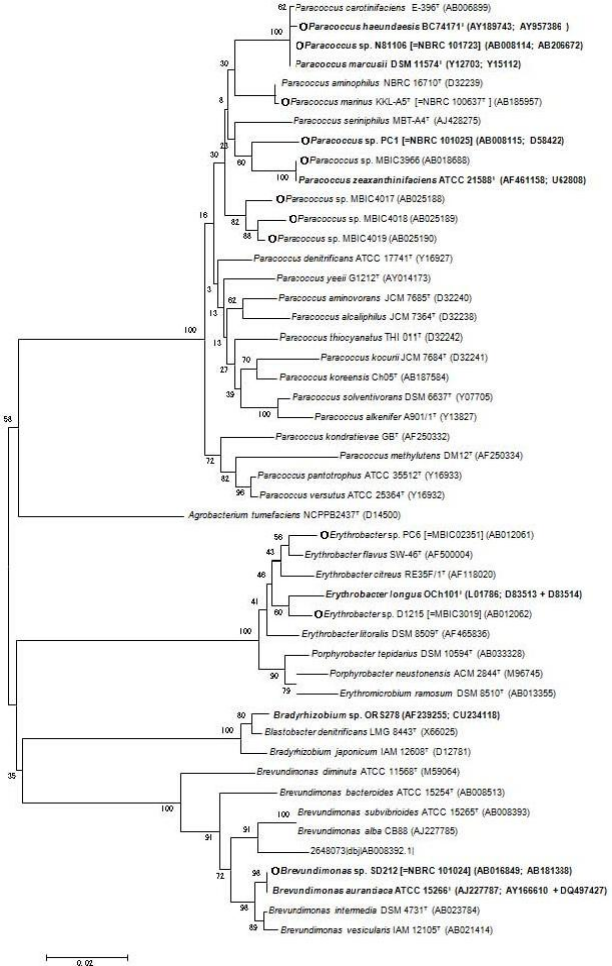
Phylogenetic positions of *Paracoccus* sp., *Erythrobacer* sp., and *Brevundimonas* sp. strains deduced from their 16S rRNA sequences. ○ represents marine bacteria. Bacterial strains, whose carotenoid biosynthesis genes were elucidated, are shown in boldface, and the second accession numbers in the parentheses shows those of carotenoid biosynthesis genes. *Paracoccus* sp. strain N81106 (MBIC01143 = NBRC 101723) and *Paracoccus* sp. strain PC1 (MBIC03024 = NBRC 101025) were formerly classified as *Agrobacterium aurantiacum* [17] and *Alacaligenes* sp. PC-1 [33], respectively. The phylogenetic tree was constructed as described [10]. The scale bar indicates a genetic distance of 0.02 (*Knuc*).

*Paracoccus* sp. strain N81106 (NBRC 101723), isolated from surface seawater near Aka island, Okinawa, Japan, was first shown to produce astaxanthin in bacteria [7,17]. This bacterium was also found to synthesize adonixanthin, adonixanthin β-d-glucoside, and astaxanthin β-d-glucoside [7,18]. *Paracoccus haeundaesis* BC74171^T^, isolated from the Haeundae Coast, Korea, was shown to produce astaxanthin mainly [19]. *Paracoccus marinus* KKL-A5^T^ (NBRC 100637^T^), isolated from coastal seawater in Tokyo Bay, Japan, was found to produce adonixanthin diglucoside predominantly [20,21]. On the other hands, a marine bacterium *Brevundimona*s sp. strain SD212 (NBRC 101024) was revealed to synthesize not only astaxanthin and adonixanthin but also their 2(2′)-hydroxylated metabolites, that is, 2-hydroxyastaxanthin ((2*R*,3*S*,3′*S*)-2,3,3′-trihydroxy-β,β-carotene-4,4′-dione), 2-hydroxyadonixanthin ((2*R*,3*S*,3′*R*)-2,3,3′-trihydroxy-β,β-caroten-4-one), erythroxanthin ((3*S*,2′*R*,3′*R*)-3,2′,3′-trihydroxy-β,β-caroten-4-one), 4-ketonostoxanthin ((2*R*,3*S*,2′*R*,3′*R*)-2,3,2′,3′-tetrahydroxy-β,β-caroten-4-one) and 2,2′-dyhydroxyastaxanthin ((2*R*,3*S*,2′*R*,3′*S*)-2,3,2′,3′-tetrahydroxy-β,β-carotene-4,4′-dione) [9]. Figure 1 shows the structures of the ketocarotenoids shown in this section and their feasible biosynthetic pathway. Figure 2 shows phylogenetic tree of the marine bacteria that produce astaxanthin and other ketocarotenoids, which were isolated in Marine biotechnology Institute (Kamaishi, Japan), along with the type strains relative to these bacteria, many of which are not marine bacteria but soil bacteria. Interestingly, the phylogenetically closest strains to the marine bacteria *Paracoccus* sp. N81106 and *Brevundimona*s sp. SD212 were soil bacteria *Paracoccus marcusii* DSM 11574^T^ [22] and *Brevundimonas aurantiaca* ATCC 15266^T^, respectively (Figure 2).

## 3. Ketocarotenoid Biosynthesis Genes

**Figure 3.**
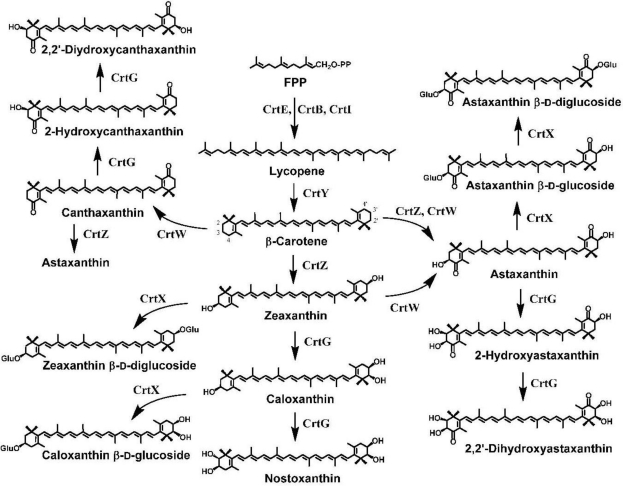
Pathway engineering for the production of functional xanthophylls using the carotenoid biosynthesis genes, *crtW*, *crtZ*, and/or *crtG*, which were isolated from the marine bacteria, *Paracoccus* sp. strain N81106 or *Brevundimonas* sp. strain SD212, in addition to the *crtE*, *crtB*, *crtI*, and *crtY* genes (and *crtX*) from *P. ananatis*.

Genes required for the biosynthesis of dicyclic carotenoids were first isolated from soil bacteria *Pantoea ananatis* (reclassified from *Erwinia uredovora*) [23] and *Pantoea agglomerans* (*Erwinia herbicola*) [24,25], which cannot produce ketocarotenoids and belong to the *Enterobacteriaceae* family of class γ*-Proteobacteria* (the same family to *Escherichia coli*). The *Pantoea* carotenoid biosynthesis genes composed a gene cluster for the synthesis of zeaxanthin β-d-diglucoside from farnesyl diphosphate (farnesyl pyrophosphate; FPP) [25–27], and comprised six genes that encode geranygeranyl diphosphate (GGPP) synthase (CrtE) [27,28], phytoene synthase (CrtB) [27,29], phytoene desaturase (CrtI) [23,30], lycopene (ψ,ψ-carotene) β-cyclase (CrtY) [23,31], β-carotene (β,β-carotene) 3-hydrocylase (CrtZ) [23], and zeaxanthin glucosyltransferase (CrtX) [23,32] (Figure 3).

These *crt* genes have widely used for complementation analysis of carotenoid biosynthesis genes isolated from other organisms, since they are functionally expressed in *E. coli* with ease [11,34–37]. The *P. agglomerans* gene cluster contained a gene encoding isopentenyl diphosphate (IPP) isomerase (Idi; type 2) [38] in addition to the six *crt* genes [39]. These seven carotenogenic (carotenoid-biosynthetic) genes were also found to exist in a carotenoid biosynthesis gene cluster of *Paracoccus* sp. strain N81106 [10,39]. This cluster included an additional gene, designated CrtW, which was elucidated to code for an enzyme responsible for ketocarotenoid formation, that is, CrtW proved to catalyze the synthesis of canthaxanthin from β-carotene by complementation analysis using recombinant *E. coli* cells that contains the *P. ananatis crtE*, *crtB*, *crtI*, and *crtY* genes [33] (Figure 3). The hydropathy and transmembrane prediction analyses indicated that CrtW from *Paracoccus* sp. N81106 contains four transmembrane domains and two other hydrophobic regions, and its topology model is very similar to those for fatty acid desaturases [40]. It should be noted that it is recalcitrant to purify active CrtW and CrtZ proteins, which both are very likely iron-dependent integral membrane proteins, from the recombinant hosts as well as the native hosts, precluding their close enzymatic characterizations.

## 4. Carotenoid 4,4′-Ketolase

**Figure 4.**
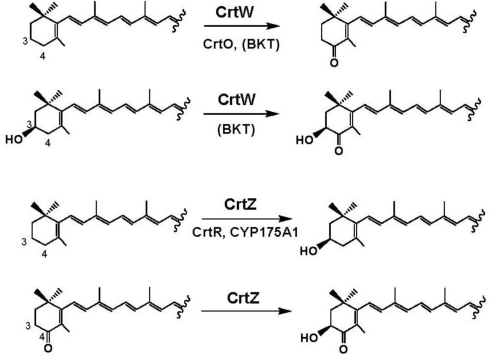
Catalytic functions of carotenoid 4,4′-ketolases (oxygenases) and carotenoid 3′3′-hydroxylases. BKT means BKT1 or BKT2 from *H. pluvialis*.

It has been revealed that only two enzymes, carotenoid 4,4′-ketolase (4,4′-oxygenase) (β-ring 4(4′)-ketolase; CrtW) and carotenoid 3,3′-hydroxylase (β-ring 3(3′)-hydroxylase; CrtZ), are sufficient to biosynthesize astaxanthin from β-carotene via eight intermediates including zeaxanthin, canthaxanthin and adonixanthin [35,40,41]. CrtW can convert not only the (un-substituted) β ring but also the 3-hydroxylated β ring into the respective 4-ketolated groups, and CrtZ can convert not only the (un-substituted) β ring but also the 4-ketolated β-ring into the respective 3-hydroxylated groups, as shown in Figure 4 [42–46]. An *in vitro* analysis with the crude enzymes of CrtW and CrtZ from the *E. coli* cells expressing the corresponding genes indicated that these enzymes are likely 2-oxoglutarate (α-ketoglutarate)-dependent dioxygenases [42].

The *crtW* genes were present not only in the above-mentioned α*-Proteobacteria* (Figure 2) but also in the marine bacterium *Algoriphagus* sp. KK10202C [4] and cyanobacterial strains such as *Anabaena* (*Nostoc*) sp. PCC 7120 and *N. punctiforme* [47,48]. These cyanobacteria produced not astaxanthin but echinenone (β,β-caroten-4-one), and 4-ketomyxol 2′-fucoside, a monocyclic carotenoid that includes the 4-ketolated β-ring [49]. Conversion efficiency to astaxanthin in several CrtWs was compared with recombinant *E. coli* cells that synthesize the carotenoid substrate zeaxanthin due to the presence of the *P. ananatis crtE*, *crtB*, *crtI*, *crtY*, and *crtZ* genes, in which each *crtW* gene from *Paracoccus* sp. N81106, *Paracoccus* sp. PC1, *Brevundimona*s sp. SD212, *Anabaena* sp. PCC7120, and *N. punctiforme* was expressed [44,46]. It was consequently shown that the *Brevundimona*s sp. SD212 CrtW, which exhibited the highest amino acid identity (96.3%) with that of the *B. aurantiaca* ATCC 15266 CrtW (accession no. AY166610), converted β-carotene to astaxanthin with the highest efficiency, along with the *P. ananatis* CrtZ [44,46]. In the case of the *Paracoccus* CrtWs, not only astaxanthin but also adonixanthin tended to accumulate, and this intermediate was difficult to be converted to astaxanthin [43,44]. The cyanobacterial CrtWs poorly converted zeaxanthin to astaxanthin via adonixanthin [46].

Two paralogous genes exhibiting significant homology to *crtW* were isolated from *H. pluvialis*, and designated *bkt* [50] or *crtO* [51]. These genes were renamed *bkt1* from *crtO* and *bkt2* from *bkt*, since “*crtO*” has been used for the other type of cyanobacterial β-ring 4(4′)-ketolase genes, as shown later [52]. The BKT1 and BKT2 enzymes are very likely to have catalytic function same to the *Paracoccus* (or *Brevundimonas*) CrtWs, considering results from the *in vitro* study on BKT2 with *E. coli* [42] and pathway engineering researches in higher plants as well as *E. coli* as the hosts [16,50,51,53].

A gene encoding a new type of β-ring 4(4′)-ketolase (named CrtO) that showed apparent homology not to CrtW-type ketolase but to CrtI-type phytoene desaturase was first found in cyanobacterium *Synechocystis* sp. strain PCC 6803 [54], which produced 3′-hydroxyechinenone (3′-hydroxy-β,β-caroten-4-one), zeaxanthin and myxol 2′-dimethyl-fucoside [55]. The *crtO* genes were also present in *Anabaena* sp. PCC 7120 [48], and an actinomycete *Rhodococcus erythropolis* and *Deinococcus radiodurans* R1 highly resistant to γ and UV radiation [56], which produced other monocyclic carotenoids, e.g., the latter strain produced deinoxanthin (2,1′-dihydroxy-3′,4′-didehydro-1′2′-dihydro-β,ψ-caroten-4-one) [1]. An *in vivo* analysis on *crtO* was performed with recombinant *E. coli* cells that synthesize the carotenoid substrate β-carotene or zeaxanthin, into which each *crtO* gene from *Synechocystis* sp. PCC 6803 and *R. erythropolis* was introduced and expressed there [57]. This result along with previous finding [48] suggested that the CrtO-type of β-ring 4(4′)-ketolases can accept only the (un-substituted) β ring(s) in β-carotene and probably in monocyclic carotenoids as the substrates (Figure 4).

## 5. Carotenoid 3,3′-Hydroxylase

The *crtZ* genes have been found not only in carotenogenic bacteria belonging to genera *Pantoea*, *Paracoccus* and *Brevundimonas*, but also in those belonging to the *Flavobacteriaceae* family [6,39]. Conversion efficiency to astaxanthin in several CrtZs was compared with recombinant *E. coli* cells that synthesize the carotenoid substrate canthaxanthin due to the presence of the *P. ananatis crtE*, *crtB*, *crtI* and *crtY* gens, and the *Paracoccus* N81106 *crtW* gene, into which each *crtZ* gene from *P. ananatis*, *Paracoccus* sp. N81106, *Paracoccus* sp. PC1, *Brevundimona*s sp. SD212, and marine bacterium strain P99-3 of the *Flavobacteriacea* family was introduced and expressed there [45]. It was consequently shown that the CrtZ enzymes from *Brevundimona*s sp. SD212 and the bacterial strain P99-3 converted β-carotene to astaxanthin with the highest and lowest efficiency, respectively, along with the *Paracoccus* N81106 CrtW [45].

On the other hands, no *crtZ* sequences have not been found in cyanobacteria, instead genes encoding a new type of β-ring 3(3′)-hydroxylases (named CrtR) that exhibited moderate homology to CrtW have been found there [58,59]. The *crtR* genes were isolated from *Synechocystis* sp. strain PCC 6803, *Anabaena* sp. PCC 7120, *Anabaena variabilis*, and *N. punctiforme* [46,58]. An *in vivo* analysis on *crtR* was performed with recombinant *E. coli* cells that synthesize the carotenoid substrate β-carotene or canthaxanthin, into which each *crtR* gene from *Synechocystis* sp. PCC 6803, *Anabaena* sp. PCC 7120, and *A. variabilis* was introduced and expressed there [46]. This result along with another result [60] indicated that the CrtR-type enzymes can hydroxylate the (un-substituted) β ring of monocyclic carotenoids such as deoxymyxol and deoxymyxol 2′-fucoside at the 3 position (Figure 4). Among them, only the *Synechocystis* sp. PCC 6803CrtR was able to convert β-carotene to zeaxanthin [46,58,60]. A thermophilic bacterium *Thermus thermophilus* HB27, which grows at temperatures above 75 °C, was found to possess another new type of β-ring 3(3′)-hydroxylase of the cytochrome P450 superfamily, named CYP175A1 [61]. The *in vivo* analysis with the gene strongly suggested that this thermostable P450 accepts only the (un-substituted) β ring of β-carotene as the substrate to form zeaxanthin [45,61].

## 6. Carotenoid 2,2′-Hydroxylase

Carotenoid 2,2′-hydroxylase (β-ring 2(2′)-hydroxylase) was first found in the marine bacterium *Brevundimonas* sp. strain SD212, and named CrtG [11]. An *in vivo* analysis on *crtG* was performed with recombinant *E. coli* cells that synthesize each carotenoid substrate (β-carotene, zeaxanthin, canthaxanthin, or astaxanthin), into which the *crtG* gene was introduced and expressed there [11]. The result indicated that the CrtG can hydroxylate the β rings substituted with 3-hydroxy and/or 4-keto groups in dicyclic carotenoids at the 2(2′)-positions (Figures 1 and 3) [11]. The *crtG* genes were also isolated from soil bacteria *Brevundimonas vesicularis* DC263 and *B. aurantiaca* ATCC 15266 [62]. The *in vivo* analysis with these genes indicated that the *B. aurantiaca* CrtG enzyme (accession no. DQ497427), which exhibited the highest amino acid identity (98.8%) to that of the *Brevundimonas* SD212 CrtG, accepted the (un-substituted) β rings of β-carotene in addition to the substituted β rings as the substrates [62]. A *crtG* gene sequence, whose encoded amino acid sequence was 41% identical to the *Brevundimonas* sp. SD212 CrtG, was found in a thermophilic cyanobacterium *Thermosynechococcus elongatus*, which synthesized 2-hydroxylated carotenoids such as caloxanthin ((2*R*,3*R*,3′*R*)-β,β-carotene-2,3,3′-triol), nostoxanthin ((2*R*,3*R*,2′*R*,3′*R*)-β,β-carotene-2,3,2′,3′-tetrol) (Figure 3), and 2-hydroxymyxol 2′-fucoside [63].

## 7. Pathway Engineering for the Synthesis of Functional Xanthophylls via the Incorporation of *crtW*, *crtZ*, and/or *crtG* Genes

Figure 3 shows xanthophylls that were produced in recombinant *E. coli* cells via the incorporation of the marine bacterial *crtW*, *crtZ*, and/or *crtG* genes along with the *Pantoea crtE*, *crtB*, *crtI*, and *crtY* genes. The recombinant *E. coli* strain that expresses the four *Pantoea crt* genes can produce β-carotene predominantly (approximately 0.2–1 mg·g^−1^ dry cell weight). The coexpression of the *crtW*, *crtZ*, and/or *crtG* genes in the β-carotene-synthesizing *E. coli* cells confer the ability to produce not only commercially important xanthophylls such as astaxanthin but also xanthophylls minor in nature (e.g., β-ring(s)-2(2′)-hydroxylated carotenoids), which are difficult to synthesize chemically. Particularly, the chemical synthesis of 2(2′)-hydroxycarotenoids are likely to be recalcitrant, due to high-density around the 1,2-positions of the β ring in these xanthophylls. We showed that the coexpression of the *Brevundimonas* sp. SD212 *crtW* gene and the *P. ananatis crtZ* gene in the β-carotene-synthesizing *E. coli* due to the presence of the four *crt* genes of *P. ananatis* resulted in predominant production of astaxanthin [44,46]. The *Paracoccus* sp. N81106 *crtW* gene was evolved by random mutagenesis to have improved activity [40]. It is also demonstrated that the coexpression of the *crtW* gene and the *crtG* gene from *Brevundimonas* sp. SD212 or from *B. aurantiaca* ATCC 15266 in the β-carotene-synthesizing *E. coli* resulted in dominant production of 2,2′-dihydroxycanthaxanthin and 2-hydroxycanthaxanthin, while the substrate canthaxanthin accumulated [11,62]. The coexpression of the *crtZ* gene and the *crtG* gene in the β-carotene-synthesizing *E. coli* resulted in predominant production of nostoxanthin along with small amounts of caloxanthin [11,62]. The coexpression of all the three genes (*crtW*, *crtZ*, and *crtG*) in the β-carotene-synthesizing *E. coli* resulted in dominant production of 2,2′-dihydroxyastaxanthin and 2-hydroxyastaxanthin [11]. When the *P. ananatis crtX* gene was coexpressed in addition to appropriate combinations of the above *crt* genes in *E. coli*, resultant *E. coli* cells were able to synthesize carotenoid-glycosides such as caloxanthin β-d-glucoside [64] and astaxanthin β-d-diglucoside [65], as shown in Figure 3.

The γ-ray-tolerant bacterium *D. radiodurans* R1 produces the monocyclic carotenoid including the 2-hydroxy-4-keto-β-ring, deinoxanthin [1]. 2,2′-Dihydroxycanthaxanthin was shown to have strong inhibitory effect against lipid peroxidation in a rat brain homogenate [11]. Such minor ketocarotenoids, which include the 2-hydroxy-4-keto-β-ring, may have beneficial effects on human health as well as anti-oxidation function, while few works are present examining their biological functions.

When carotenoid biosynthesis genes starting from the utilization of FPP are introduced in *E. coli*, as above-mentioned, amounts of carotenoids produced with the recombinant *E. coli* cells are far from the practical use, which was difficult to exceed 1 mg·g^−1^ dry weight. In order to overcome this problem, many pathway engineering researches in *E. coli* have been performed for increasing intracellular concentration of FPP (e.g., recently reviewed [66,67]). For example, the coexpression of the *idi* (type 1) gene from *H. pluvialis*, *Xanthophyllomyces dendrorhous* (renamed from *Phaffia rhodozyma*), or *Saccharomyces cerevisiae*, as well as the *idi* (type 2) from *Streptomyces* sp. strain CL190, was shown to be effective to increase FPP content [68,69]. The introduction of heterologous mevalonate pathway genes in *E. coli* along with an *idi* (type 2) gene has been described to efficiently improve the productivity of carotenoids or sesquiterpenes that are synthesized from FPP [69–73]. For example, Yoon *et al.* [73] produced 22 mg·g^−1^ dry cell weight of lycopene in 72 h using such mevalonate-pathway-engineered *E. coli* cells. On the other hand, production of lycopene reached high levels (near to 20 mg·g^−1^ dry cell weight) in 24-h batch flask culture in pathway-engineered *E. coli*, which reflected results of multi-dimensional gene target search or gene-knockout analysis [74]. These finding should be applied to efficient production of the above-mentioned functional xanthophylls with *E. coli* cells.

Pathway engineering researches in higher plants have also been performed for efficient production of astaxanthin, which utilized the marine bacterial *crtW* genes from *Paracoccus* sp. N81106 or *Brevundimonas* sp. SD212, or the *H. pluvialis bkt1* or *bkt2* genes, as reviewed [16,39,53]. For example, the *Brevundimonas* sp. SD212 *crtW* and *crtZ* genes, whose nucleotide sequence is modified to codon usage of higher plants, were successfully overexpressed in the chloroplasts of tobacco plants (*Nicotiana tabacum*), and astaxanthin level produce there reached 5.44 mg·g^−1^ dry weight (74% of total carotenoids) [75].

## 8. Conclusions

This review has focused on the carotenoid β-ring-modifying enzymes, CrtW, CrtZ and CrtG, derived from the marine bacteria of the α-*Proteobacteria* class, and pathway engineering for the production of xanthophylls in *E. coli*, using these enzyme genes. Such pathway engineering researches are performed towards efficient production not only of commercially important xanthophylls such as astaxanthin, but also of xanthophylls minor in nature, which are difficult to synthesize chemically, and expected to have beneficial effects on human health as well as anti-oxidation function.
